# Epidemiological description of fire blight introduction patterns to Central Asia and the Caucasus region based on CRISPR spacer typing and genome analysis

**DOI:** 10.1186/s42483-024-00283-4

**Published:** 2024-11-20

**Authors:** Fabio Rezzonico, Saykal Bobushova, Dali Gaganidze, Mahabat Konurbaeva, Sergey Mukhanov, Sara Jordan, Tinatin Sadunishvili, Nataliya Drenova, Theo H. M. Smits, Tinatin Doolotkeldieva

**Affiliations:** 1 Environmental Genomics and Systems Biology Research Group, Institute for Environment and Natural Resources, Zürich University for Applied Sciences (ZHAW), 8820 Wädenswil, Switzerland; 2 Kyrgyz-Turkish Manas University, 720044 Bishkek, Kyrgyzstan; 3 Sergi Durmishidze Institute of Biochemistry and Biotechnology, Agricultural University of Georgia, 0159 Tbilisi, Georgia; 4 All-Russian Plant Quarantine Centre, Bykovo, Ramenskoe, Moscow Russian Federation 140150; 5 Present Address: Plant Protection Centre, Kyrgyz National Agrarian University after the name K.I. Skraybin, Bishkek, Kyrgyzstan

**Keywords:** *Erwinia amylovora*, CRISPR repeat regions, Genotyping, Genetic diversity, *Malus sieversii*

## Abstract

In the last two decades, fire blight has progressively spread eastward from Europe and the Mediterranean area to several pome-fruit producing regions of Asia. Its causative agent, the bacterial pathogen *Erwinia amylovora*, was detected in several new countries, including Georgia, Kyrgyzstan, and Kazakhstan. In the latter two states, the disease creates a threat not only to the commercial apple and pear production, but also to the wild *Malus* and *Pyrus* species that constitute the basis of the local forest ecosystems. In this study, we investigated the genetic diversity of the pathogen in Central Asia and the Caucasus region utilizing CRISPR Repeat Regions (CRRs) genotyping and genome sequencing, with the aim to understand its dissemination patterns across the continent. Genome sequence analysis revealed that all strains from these two regions exclusively derived from the archetypal CRR1 genotype A. Our analysis revealed three main *E. amylovora* clades in Central Asia, with distinct yet partial overlapping geographical distributions. Genomic relationships among isolates indicate that Central Asian strains are genetically closest to those from the Persian region and the Middle East, while the Georgian population is genetically more distant and can align with strains from the Volga District in southern Russia and the Eastern Mediterranean area. Notably, this study also includes strains from the first confirmed occurrences of fire blight in Uzbekistan, Tajikistan, and China. Our findings highlight the importance of phylogenetic analysis and genome sequencing in understanding the phytopathogen epidemics and protecting key agricultural species and the genetic resources of their wild counterparts in the forest.

## Background

Central Asia is the geographic origin of domesticated apple (*Malus*) and pear (*Pyrus*) species. Unlike anywhere else in the world, in Kyrgyzstan and Kazakhstan, wild apple and pear are dominant forest species in mid-altitude mountainous regions, thereby representing a critical foundation for whole ecosystems of plants, insects, and animals. The genetic diversity in these local ancestral *Malus* and *Pyrus* spp. (many of them included in the International Union for Conservation of Nature (IUCN) Red List of Threatened Species) represents an invaluable and irreplaceable germplasm resource that is now endangered by the expansion of fire blight in the region (Maltseva et al. 2023).

*Erwinia amylovora*, the causative agent of fire blight, is native to North America and was imported to Western Europe and the Mediterranean Area during the 1950s, from where it spread from west to east in the following decades (Norelli et al. 2003; Kurz et al. 2021). The disease reached Central Asia in 2008, when it was nearly simultaneously recorded in Kyrgyzstan and in Kazakhstan (Drenova et al. 2012; Doolotkeldieva and Bobusheva 2016). The introduction of the pathogen was facilitated by the massive import of seedlings from countries with recorded fire blight history along with weak quarantine controls (Djaimurzina et al. 2014; Umiraliyeva et al. 2021; Maltseva et al. 2023). The disease subsequently spread to several fruit growing areas in both countries, mainly affecting plants in orchards and private gardens (Doolotkeldieva et al. 2019; Kurz et al. 2021). In Georgia, fire blight first appeared in 2016 in the Mtskheta-Mtianeti region before rapidly propagating to all pome-fruit growing zones in the East of the country (Gaganidze et al. 2021).

The genetic diversity within *E. amylovora* in isolates outside North America is extremely low (Rezzonico et al. 2011), with all Eurasian and North African isolates belonging exclusively to one of the four major clades characterizing the group of *Amygdaloideae*-infecting strains. This so-called Widely-Prevalent (WP) clade displays a remarkably low variability resulting in a similarity exceeding 99.99% at genome level (Mann et al. 2013; Parcey et al. 2020), which makes the investigation of genetic diversity arduous. Meaningful epidemiological studies can thus only be performed by whole-genome sequencing (WGS) or by resorting to the characterization of highly variable regions of the genome such as those represented by the spacers in the CRISPR repeat regions (CRRs). CRRs are part of the CRISPR/Cas adaptive immune system that protect bacteria against foreign DNA and their analysis can also provide valuable chronological evidence about the succession of genotypes within a defined geographical area (Rezzonico et al. 2011; McGhee and Sundin 2012; Tancos and Cox 2016; Mendes et al. 2021; Wallis et al. 2021; Parcey et al. 2022). Based on a single CRR1 spacer duplication distinguishing the two archetypal European lineages, we previously hypothesized that there have been at least two distinct introduction events of fire blight from North America to Europe (Bühlmann et al. 2014; Kurz et al. 2021) and that only isolates belonging to the so-called A-derived genotype were present in Kyrgyzstan, Kazakhstan, and Georgia (Doolotkeldieva et al. 2021; Gaganidze et al. 2021; Kurz et al. 2021; Sadunishvili et al. 2024). In this study, we expand our earlier PCR typing approaches of *E. amylovora* by examining the CRRs and the genome sequences of a broader set of isolates collected not only in the three countries mentioned above, but also originating throughout the Eurasian continent and the Mediterranean basin during the last half century.

## Results

### 2020–2022 survey results in Kyrgyzstan and Georgia

The monitoring campaign in summer 2021 represented the first effort to identify fire blight foci in the orchards of the Talas region, Kyrgyzstan. Several villages in the Bakai-Ata district were inspected and isolates of *E. amylovora* were recovered from plants showing indications of fire blight in Ozgorush, Ak Dobo, and Tegirmen Sai. Symptoms were mostly noticeable in tall and semi-dwarf trees, especially in pears, but also in seedlings in nurseries. As the survey occurred in autumn during the ripening period of the winter varieties, indications of infections were mainly evident on the apical branches, on the unfallen scorched leaves and on the fruits. During the same period and in 2022, more isolates were collected from the Chui Valley, the region of Jalal-Abad and the surroundings of lake Issyk-Kul, which were all already sampled in earlier years. A total of 40 isolates were obtained from 133 samples.

In Georgia, the presence of *E. amylovora* was confirmed in four regions (Mtskheta-Mtianeti, Shida Kartli, Kvemo Kartli, and Kakheti) where it was already detected in the period 2016–2018 (Gaganidze et al. 2021), thus showing the persistence of the disease in the eastern part of the country. Fire blight symptoms were observed in all cultivated pomaceous plants, even though most of the positive probes were isolated from apple trees. Here, 52 isolates were obtained (Sadunishvili et al. 2024).

**Fig. 1:**
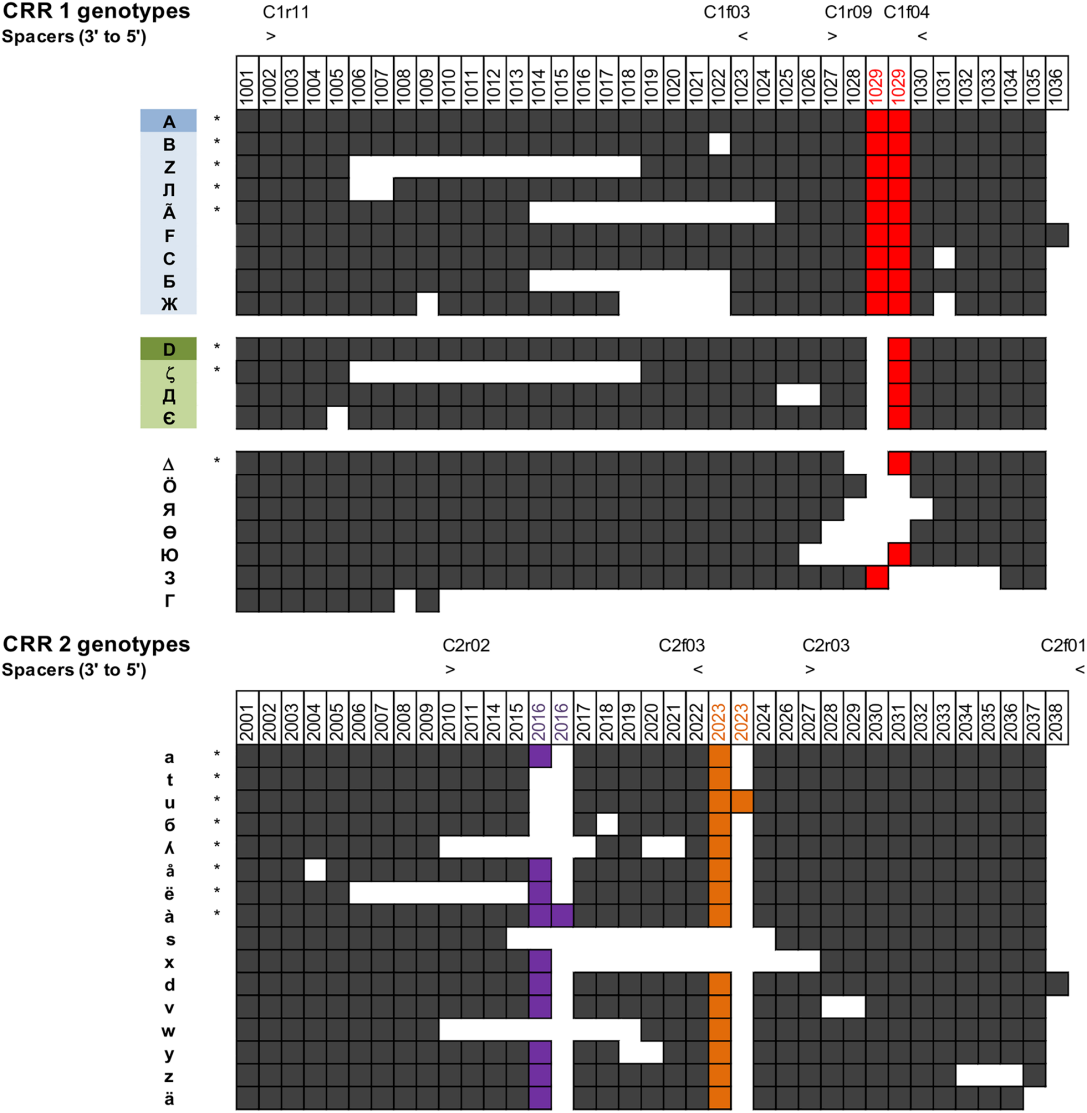
Spacer organization of the *E. amylovora* CRR1 and CRR2 genotypes observed in this study. The arrays are oriented in the 3′-to-5′ direction with the newest spacers next to the leader sequence on the right side of the picture. Numbering of the spacers is coherent with that proposed by Rezzonico et al. (2011), while duplications of identical spacers are highlighted in color. The position and direction of the primers used for preliminary PCR screening is indicated by the major (>) and minor (<) signs above the respective arrays next to the primer names. A- and D-derived CRR1 genotypes, as they would be detected using the PCR approach proposed by Kurz et al. (2021), are highlighted in blue and green colors, respectively. Genotypes retrieved in Central Asia are indicated by an asterisk (*)

Overall, ten Kyrgyz and five Georgian isolates from the 2021–2022 seasons were selected for WGS on the basis of the PCR amplicon length polymorphism previously observed in the CRR1 and CRR2 using primers C1f04/C1r09 (Kurz et al. 2021), C1f03/C1r11, and C2f03/C2r02 (Doolotkeldieva et al. 2021) or C2f01/C2r03 (Gaganidze et al. 2021; Sadunishvili et al. 2024) (Fig. 1).

### Analysis of the CRISPR regions

**Fig. 2:**
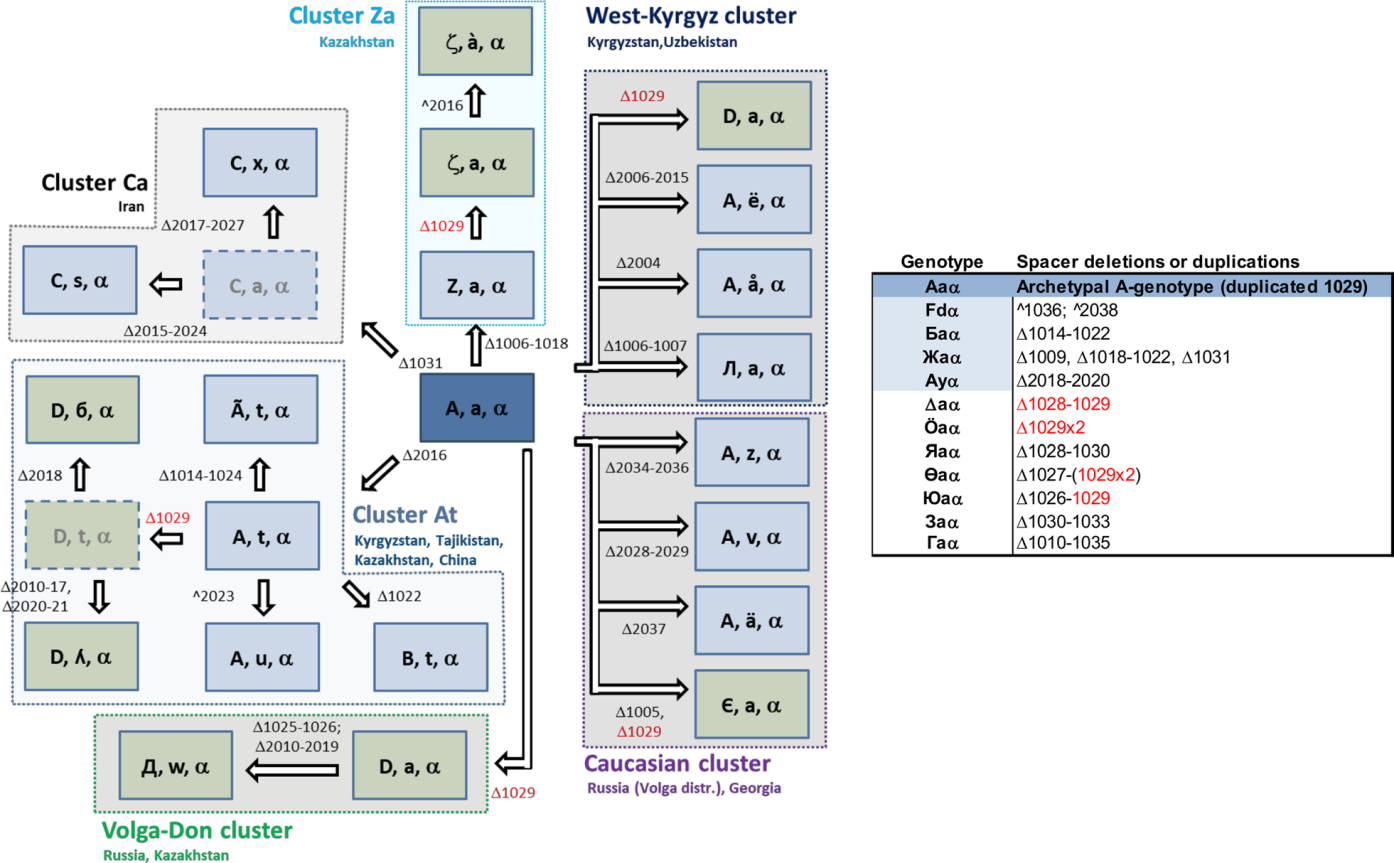
Relationship among the different CRISPR genotypes of *E. amylovora* analyzed in this study in terms of spacer losses (Δ) or duplications (^). Six main clusters with discrete geographical distribution, all originating from archetypal genotype (A, a, α), could be identified (left). Deletions of spacer 1029 (Δ1029) cause an apparent reversion from an A-derived (blue boxes) to a D-derived (green boxes) or a null genotype, if analyzed with the PCR approach proposed by Kurz et al. (2021). Putative intermediate genotypes not found in this work are represented by dashed boxes. Genotypes that could not be directly assigned to any group, as well as their modification with respect to archetypal genotype (A, a, α) are shown in a separate box (right)

A total of 35 isolates from five different Central Asian countries as well as nine isolates from Georgia spanning the periods 2011–2022 and 2016–2022, respectively, were included in the CRISPR repeat analysis based on the availability of their genomes. Forty-two previously published genomes of older isolates from Europe, the Mediterranean area, and the Middle East, as well as one reference genome from the United States, were included in the study for comparison (Table 1). Overall, 20 and 16 different CRR1 and CRR2 genotypes were identified, respectively. The number of spacers varied between 8 and 37 in CRR1 or 24 and 35 in CRR2 (Fig. 1). As expected, no variability was observed in the CRR4 genotype that consistently displayed the five spacers that are typical for the three main groups of the *Amygdaloideae*-infecting *E. amylovora* subtype (Rezzonico et al. 2011; McGhee and Sundin 2012; Parcey et al. 2022). The analysis of the CRRs resulted in a total of 32 unique genotypes that were initially assigned to two separate groups depending on the presence (A-derived genotypes) or absence (D-derived genotypes) of the duplication of spacer 1029 in CRR1, as previously described (Kurz et al. 2021). Genotypes in which spacer 1029 was not present, or that presented spacers deletion immediately upstream or downstream of a single-copy spacer 1029, were assigned to a third group under the rationale that it was not possible to determine if they originally contained one or two copies of that spacer prior to the deletion. The most parsimonious relationship in terms of spacer deletions or duplications between the different genotypes is inferred in the next section and illustrated in Fig. 2.

**Table 1:** List of isolates used in this study divided by method used to retrieve their genome or CRRs sequences

Isolate	Country	District, village	Region	Host	Year	Genotype	References	
*Whole-genome sequencing*								
Chui-3	KG	KashkaSuu	Chui Valley	*P. communis* var. *Talgarskaya*	2021	(A, u, α)	This work	
GE6053	GE	Sighnaghi, Jugaani	Kakheti	*M. domestica*	2021	(A, a, α)	This work	
GE6163	GE	Sighnaghi, Jugaani	Kakheti	*M. domestica*	2021	(A, z, α)	This work	
GE6931	GE	Khashuri, Gomi	Shida Kartli	*Cydonia* sp.	2021	(A, z, α)	This work	
GE13011	GE	Marneuli, Kachagani	Kvemo Kartli	*M. domestica*	2022	(A, ä, α)	This work	
GE13133	GE	Marneuli, Kachagani	Kvemo Kartli	*M. domestica*	2022	(A, z, α)	This work	
IS-9	KG	Tong, Bokonbaev	Issyk-Kul North	*P. communis* var. *Talgarskaya*	2021	(A, t, α)	This work	
IS-19	KG	Barskoon	Issyk-Kul South	*P. communis* var. *Talgarskaya*	2021	(A, t, α)	This work	
KazE4	KZ	Resul	Zhambyl	*P. communis*	2014	(A, t, α)	This work	
KazE6	KZ	Sayran	South-Kazakhstan	*M. domestica*	2014	(ζ, a, α)	This work	
KazE7	KZ	Sayran	South-Kazakhstan	*M. domestica*	2014	(ζ, à, α)	This work	
KazE9	KZ	№173.2.1	N.A.	N.A.	2013	(Δ, a, α)	This work	
KazE15	KZ	№87	Almaty reg.	*Chaenomeles japonica*	2015	(A, t, α)	This work	
KazE17	KZ	Enbekshikazakh	Almaty reg.	*Malus sieversii*	2016	(D, a, α)	This work	
KazE19	KZ	n.a.	Almaty reg.	*M. domestica* var. *Aport*	2017	(A, t, α)	This work	
KG03-22	KG	YshelieSakMazar, Arslonbob	Jalal-Abad	*C. turkestanica*	2022	(A, å, α)	This work	
KG04-22	KG	Oogantalaa, Arslonbob	Jalal-Abad	*P. communis* var. *Talgarskaya*	2022	(A, å, α)	This work	
KG09-22	KG	Kemin district	Issyk-Kul North	*M. domestica*	2022	(D, ʎ, α)	This work	
KG49	KG		Chui Valley	*M. domestica*	2013	(A, t, α)	This work	
KZ89	KZ		Almaty reg.	*M. domestica*	2011	(Z, a, α)	This work	
TaE1	TJ	Shahrinav	Tajikistan	*M. domestica*	2017	(B, t, α)	This work	
TL-13.1	KG	Bakai-Ata, Ozgorush	Talas	*M. domestica* var. *Prevoshodnaya*	2021	(D, б, α)	This work	
TL-46	KG	Bakai-Ata, Ak Dobo	Talas	*P. communis* var. *Talgarskaya*	2021	(D, б, α)	This work	
TL-63	KG	Bakai-Ata, Tegirmen Sai	Talas	*P. communis* var. *Talgarskaya*	2021	(Л, a, α)	This work	
UzE2	UZ	Samarkand	Uzbekistan	*M. domestica*	2017	(D, a, α)	This work	
UzE3	UZ	Samarkand	Uzbekistan	*M. domestica*	2017	(A, a, α)	This work	
WP-13	KG	Nooken, Tockool-Ata	Jalal-Abad	*Pyrus korzhinski*	2021	(A, ë, α)	This work	
*CRRs sequencing*								
GE01	GE	Kareli	Shida Kartli	*M. domestica*	2016	(A, z, α)	(Gaganidze 2021)	
GE02	GE	Marneuli	Kvemo Kartli	*M. domestica*	2018	(A, z, α)	(Gaganidze 2021)	
GE03	GE	Marneuli	Kvemo Kartli	*M. domestica*	2018	(A, a, α)	(Gaganidze 2021)	
GE04	GE	Gori	Shida Kartli	*M. domestica*	2018	(A, a, α)	(Gaganidze 2021)	
KG44	KG		Chui Valley	*M. domestica*	2013	(A, t, α)	(Doolotkeldieva 2021)	
KG52	KZ		Chui Valley	*P. communis*	2013	(A, t, α)	(Doolotkeldieva 2021)	
KTMU-15	KG	JetiOguz, Saruu	Issyk-Kul South	*P. communis* var. *Talgarskaya*	2018	(A, t, α)	(Doolotkeldieva 2021)	
KZ18	KZ		Zhambyl	*P. communis*	2013	(Z, a, α)	(Doolotkeldieva 2021)	
KZ74	KZ		Almaty reg.	*M. domestica*	2013	(A, t, α)	(Doolotkeldieva 2021)	
KZ79	KZ		Almaty reg.	*M. domestica*	2013	(A, t, α)	(Doolotkeldieva 2021)	
*Genome sequence previously available*								
241/07	IL			*P. communis*	2007	(Я, a, α)	(Parcey 2020)	
99-east-3-1	CN	Bayingolin	Xinjiang	*P. sinkiangensis*	2021	(Ã, t, α)	(Fei et al. 2023b)	
BPIC847	GR		Arcadia	*Pyrus* sp.	1984	(A, a, α)	(Bühlmann 2015)	
CFBP 1232^T^	UK			*P. communis*	1959	(A, a, α)	(Mann 2013)	
CFBP 1252	UK			*P. communis*	1958	(A, a, α)	(Bühlmann 2015)	
CFBP 1367	FR			*Crataegus oxyacantha*	1972	(A, a, α)	(Bühlmann 2015)	
CFBP 1430	FR			*C. oxyacantha*	1972	(A, a, α)	(Smits et al. 2010)	
CFBP 2586	IE			*Pyracantha* sp.	1986	(A, a, α)	(Bühlmann 2015)	
CFBP 3020	NL			*P. communis*	1981	(A, a, α)	(Bühlmann 2015)	
CFBP 3098	IL	Masluk	Negev	*M. domestica*	1987	(D, a, α)	(Bühlmann 2015)	
CFBP 3860	HU			*Malus* sp.	1996	(A, a, α)	(Bühlmann 2015)	
CPBF 1309	PT		Alcobaça	*P. communis*	2010	(D, a, α)	(Bühlmann 2015)	
E-2	BY		Myadzel	*Malus* sp.	2007	(D, a, α)	(Lagonenko et al. 2008)	
Ea1/79	DE			*Cotoneaster* sp.	1979	(D, a, α)	(Parcey 2020)	
Ea1	IR		Loristan	*Pyrus* sp.	2009/10	(C, s, α)	(Bühlmann 2015)	
Ea2	IR		Loristan	*Pyrus* sp.	2009/10	(З, a, α)	(Bühlmann 2015)	
Ea32	IR		East-Azerbaizjan	*Cydonia* sp.	2009/10	(A, a, α)	(Bühlmann 2015)	
Ea33	IR		Semnan	*Cydonia* sp.	2009/10	(C, x, α)	(Bühlmann 2015)	
Ea169	IL			*P. communis*	N/A	(Ɵ, a, α)	(Parcey 2020)	
Ea209	IL			*P. communis*	1997	(A, a, α)	(Bühlmann 2015)	
Ea263	IL			*C. oblonga*	1997	(F, d, α)	(Bühlmann 2015)	
Ea326	CH		Aargau	*Pyracantha* sp.	1990	(D, a, α)	(Bühlmann 2015)	
Ea4/82	EG			*P. communis*	1982	(A, a, α)	(Bühlmann 2015)	
Ea650	PL			*Crataegus monogyna*	1986	(D, a, α)	(Parcey 2020)	
Ea779/01	AT		Vorarlberg	*Pyrus* sp.	2001	(D, a, α)	(Bühlmann 2015)	
EaA-11	LB			*M. domestica*	N/A	(Ю, a, α)	(Parcey 2020)	
EaB-110	LB			*M. domestica*	N/A	(Ö, a, α)	(Parcey 2020)	
EaK1	IR		Karaj	*Malus* sp.	2004	(A, a, α)	(Bühlmann 2015)	
FEa9	PL		Wojewodztwo Mazowiecki	*Pyracantha* sp.	2011	(D, a, α)	(Bühlmann 2015)	
FEa10	KZ	Enbekshikazakh	Almaty reg.	*M. domestica*	2012	(A, u, α)	(Bühlmann 2015)	
FEa11	KZ		Almaty reg.		2012	(Z, a, α)	(Bühlmann 2015)	
KBE1	RU		Kabardino-Balkaria	*Cydonia* sp.	2009	(Б, a, α)	(Bühlmann 2015)	
KE9	RU		Kaliningrad	*Crataegus* sp.	2003	(D, a, α)	(Bühlmann 2015)	
KG58	KG		Chui Valley	*M. domestica*	2013	(A, t, α)	(Bühlmann 2015)	
KZ03	KZ		Zhambyl	*P. communis*	2013	(Z, a, α)	(Bühlmann 2015)	
KZ29	KZ		South-Kazakhstan	*M. domestica*	2013	(Z, a, α)	(Bühlmann 2015)	
Leb A3	LB			*M. domestica*	1998	(D, a, α)	(Bühlmann 2015)	
Leb B66	LB			*C. oblonga*	1998	(A, a, α)	(Bühlmann 2015)	
MOE1	MD		Moldavia	*M. domestica*	2007	(A, y, α)	(Bühlmann 2015)	
S618-2-2	CN	Bayingolin, Korla	Xinjiang	*P. sinkiangensis*	2021	(A, t, α)	(Fei et al. 2023a)	
SAE3	RU		Saratov	*M. domestica*	2012	(A, v, α)	(Bühlmann 2015)	
SE1	RU		Samara	*M. domestica*	2008	(A, a, α)	(Bühlmann 2015)	
SE11	RU		Samara	*M. domestica*	2012	(D, a, α)	(Bühlmann 2015)	
TE4	RU		Tambov	*M. domestica*	2007	(Д, w, α)	(Bühlmann 2015)	
Tk86	TK		Bursa	*Cydonia* sp.	2007	(Ж, a, α)	(Bühlmann 2015)	
Tk119	TK		Izmir	*P. communis*	2011	(Г, a, α)	(Bühlmann 2015)	
Tk159	TK		Zonguldak	*Cydonia* sp.	2012	(A, a, α)	(Bühlmann 2015)	
VGE1	RU		Volgograd	*Cydonia* sp.	2010	(Є, a, α)	(Bühlmann 2015)	
VRE4	RU		Voronezh	*P. communis*	2010	(D, a, α)	(Bühlmann 2015)	
WSDA87-73	US		Washington	*M. domestica*	N/A	(D, a, α)	(Zeng et al. 2018)	

Country codes: AT, Austria; BY, Belarus; CH, Switzerland; CN, China; DE, Germany; EG, Egypt; FR, France; GE, Georgia; GR, Greece; HU, Hungary; IE, Ireland; IL, Israel; IR, Iran; KG, Kyrgyzstan; KZ, Kazakhstan; LB, Lebanon; MD, Moldova; NL, The Netherlands; PL, Poland; PT, Portugal; RU, Russia; TJ, Tajikistan; TK, Turkey; UK, United Kingdom; US, United States; UZ, Uzbekistan

### Geographical distribution of CRISPR genotypes in Central Asia

**Fig. 3:**
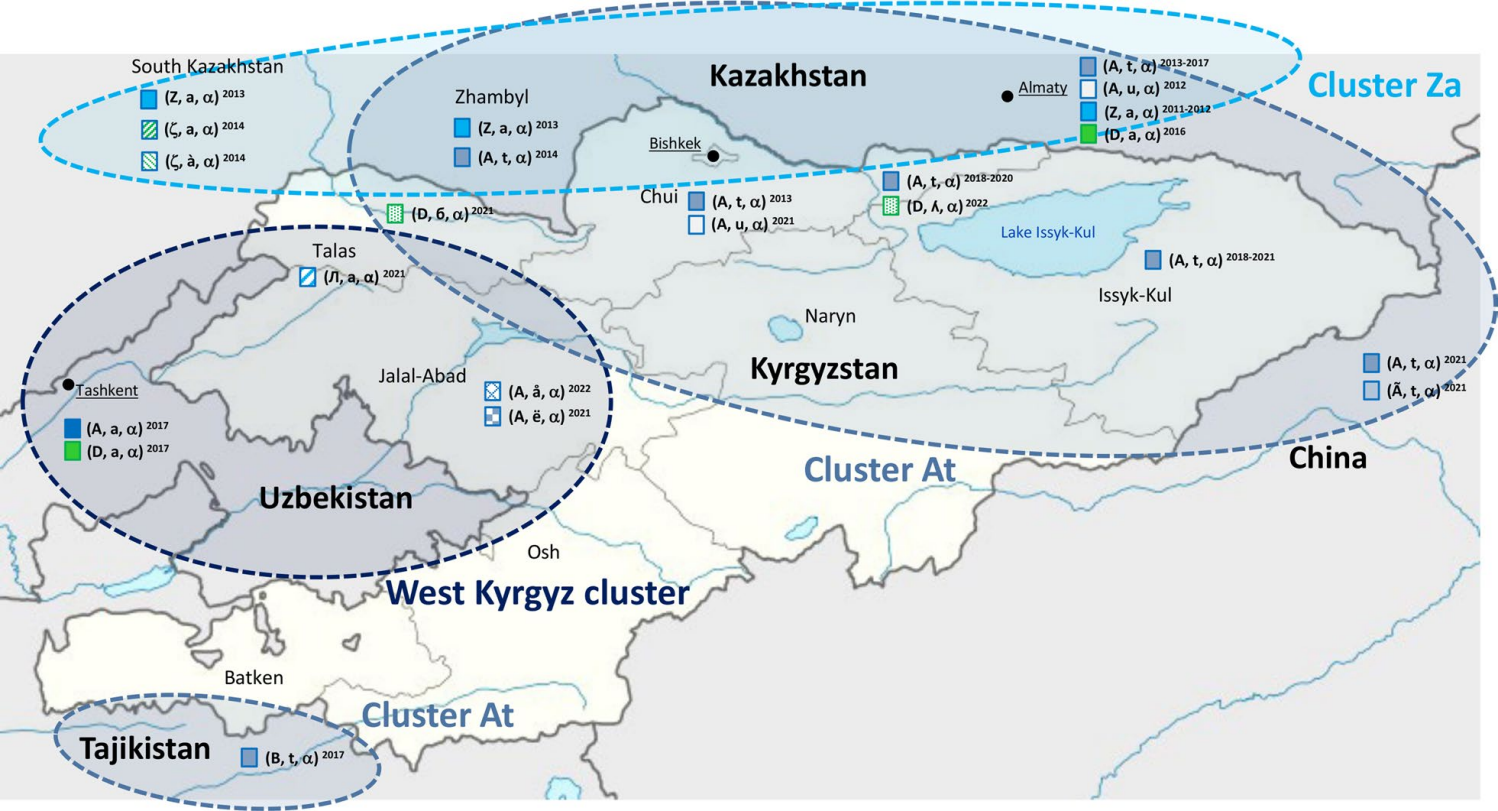
Distribution of different CRISPR genotypes of *E. amylovora* in Central Asia. Isolates were clustered according to the most parsimonious relationship among genotypes described in Fig. 2. Only isolates with completely sequenced CRR were taken into consideration to generate the map. The exact geographical origin of isolate KazE9 (Δ, a, α) is not determined

Archetypal genotype (A, a, α), as in type strain CFBP 1232^T^, has the most complete CRRs among all isolates belonging to the widely prevalent (WP) group and was originally introduced in the United Kingdom in 1957 from the US east coast (Rezzonico et al. 2011; Parcey et al. 2020). Due to these characteristics, it is thus considered one of the two founder genotypes in Europe along with archetypal genotype (D, a, α), which does not display the duplication of spacer 1029 and was first detected in strain Ea 1/79 in 1979 in Germany (Kurz et al. 2021). Preliminary PCR screening with primers C1f04/C1r09 (Kurz et al. 2021) suggested that some of the new isolates obtained from Central Asia in this study apparently carried a D-derived genotype, which was not previously observed in the region. In this study, both genotypes (A, a, α) and (D, a, α) were observed in the two strains retrieved in 2017 on *Malus domestica* from the Samarkand region in Uzbekistan and, for genotype (D, a, α), in one strain recovered in 2016 from the Almaty region in Kazakhstan (Fig. 3). All other isolates from Central Asia displayed CRISPR patterns apparently derived from one of the two archetypal genotypes mentioned above through spacer loss.

(A, a, α)-derived genotype (A, t, α), resulting from the deletion of spacer 2016 (Fig. 2), was geographically and temporally the most extensively distributed subpopulation of *E. amylovora* in Central Asia. It was detected both in the Zhambyl and Almaty regions in Kazakhstan, as well as in the Chui Valley and in the region surrounding lake Issyk-Kul in north Kyrgyzstan. This is also the genotype of strain S618-2-2 on *Pyrus sinkiangensis*, which was reported in 2021 from the Xinjiang region in China (Fei et al. 2023a) (Fig. 3). Isolates carrying this genotype can be tracked back to 2013 both in Kyrgyzstan and in Kazakhstan, spanning most of the recorded history of fire blight in Central Asia. Possible direct derivatives of the (A, t, α) pattern are genotypes (A, u, α) and (B, t, α), additionally displaying the duplication of spacer 2023 and the deletion of spacer 1022, respectively, as well as the genotype (Ã, t, α) of the second Chinese strain 99east-3-1 (Fei et al. 2023b), which carries an extensive deletion of spacers 1014–1024 in CRR1 (Fig. 2). Genotype (A, u, α) is present both in the Chui Valley in Kyrgyzstan and in the Almaty region in Kazakhstan, whereas genotype (B, t, α) characterizes strain TaE1, the only isolate available from Tajikistan so far (Fig. 3). Within the same cluster, the presence of seemingly D-derived genotypes could be confirmed in isolates from the Talas region and the surrounding area of lake Issyk-Kul in Kyrgyzstan (Fig. 3), where genotypes (D, б, α) and (D, ʎ, α) were detected, respectively. Beside the loss of one of the two copies of spacer 1029, these two genotypes additionally displayed the loss of one or more spacers in the CRR2 compared to genotype (A, t, α) (Fig. 2).

Genotype (Z, a, α) is also a derivative of archetypal genotype (A, a, α), in which the entire region of CRR1 spanning spacers 1006 to 1018 was lost (Fig. 2). This genotype, present in all southern regions of Kazakhstan, i.e., South Kazakhstan, Zhambyl, and Almaty regions (Fig. 3), was detected in the latter region already in 2011, making it the earliest genotype characterized in Central Asia. Two variations of genotype (Z, a, α) were found in South Kazakhstan, both originating from the deletion of one of the two duplicated spacers 1029 that are typical for archetypal genotype A (Kurz et al. 2021). On top of this first modification, here denoted with the genotype (ζ, a, α), the subsequent duplication of spacer 2016 resulted in genotype (ζ, à, α) (Fig. 2). Despite their probable origin as (Z, a, α)-genotype derivatives, both associated strains KazE6 and KazE7 yield a 215-bp amplicon compatible with a D-derived lineage using primer pair C1f04/C1r09 as proposed by Kurz et al. (2021).

Genotypes (A, å, α) and (A, ë, α), presenting the deletions of spacer 2004 and of spacer array 2006–2015, respectively, were only found in isolates from the Arslonbob forest in the Jalal-Abad region in Kyrgyzstan (Fig. 3), while genotype (Л, a, α), resulting from the deletion of CRR1 spacers 1006–1007, was identified in the Talas region. All genotypes from this group are most likely directly derived from archetypal genotype (A, a, α) (Fig. 2).

Overall, three main *E. amylovora* clusters could be identified in Central Asia that showed a discrete but partially overlapping geographical distribution (Fig. 3). All the genotypes within these groups were shown to be directly derived from archetypal genotype (A, a, α), although some of them displayed the deletion of one of two copies of spacer 1029, thus apparently reverting to a D-derived genotype in the PCR approach used for preliminary screening (Kurz et al. 2021).

### Genomic relationships among isolates in Central Asia and in Georgia

**Fig. 4:**
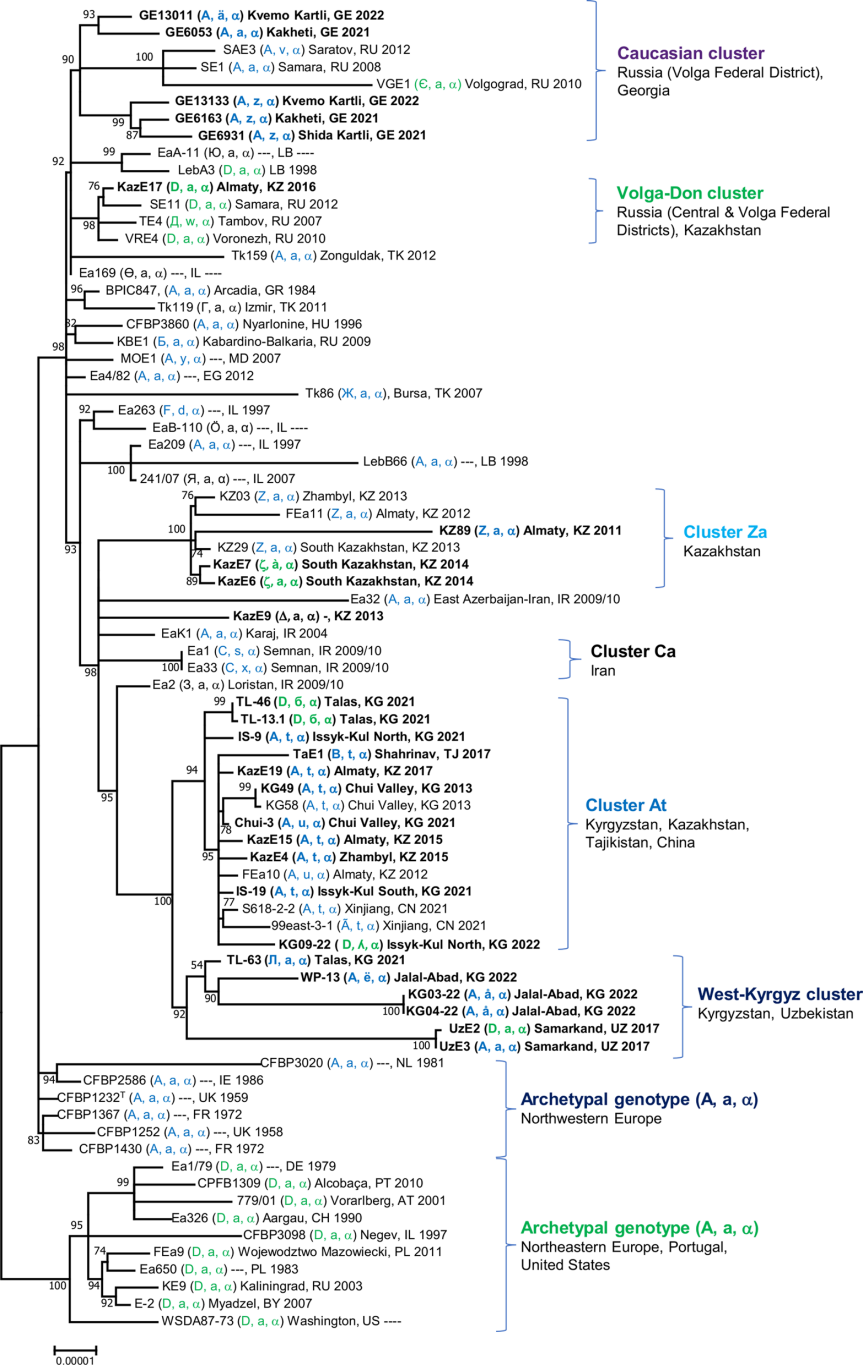
Approximately-maximum-likelihood phylogenetic tree for the 77 genomes of *E. amylovora* build out of a core of 2738 genes per genome. The strains that were sequenced for this work are marked in bold. The core has 903,563 AA-residues per genome. The scale distance at the bottom (0.00001 substitutions per site) corresponds to around 40 AA substitutions per genome. Significant local support values, determined using the Shimodaira-Hasegawa test, are indicated at branch points

To verify the results obtained with the analysis of the CRR, a phylogenetic core genome tree (Fig. 4) was calculated using the available genome sequences of 77 of the 87 strains included in Table 1. A group of eight European strains from the years 1979–2011 was included in the analysis as reference representing the archetypal genotype (D, a, α) (Rezzonico et al. 2011; Kurz et al. 2021), together with a strain each from the United States and from Israel (Fig. 4). These ten strains formed a discrete clade on the genome tree alongside another group of older European strains from the period 1959–1986 belonging to the archetypal genotype (A, a, α), thus corroborating the previous hypothesis of two initial separate introduction events from North America. However, despite the apparent presence of both A- and D-derived genotypes among the Asian isolates, genomic analysis clearly indicated that they were all derived from the original European lineage characterized by archetypal genotype (A, a, α), rather than from archetypal genotype (D, a, α) (Fig. 4). This result implies that the loss of one of the two copies of spacer 1029 occurred in several independent instances during the eastward expansion of fire blight, such as in the Volga-Don cluster constituted by three strains from the Central and Volga Federal Districts in Russia and one from the Almaty region in Kazakhstan that contains the (D, a, α) genotype and its variant (Д, w, α) (Fig. 4).

Genome analysis also corroborated the shift from the (Z, a, α) to the (ζ, a, α) genotype in South Kazakhstan that was hypothesized, based on an extended deletion in the CRR1 region (Fig. 2). According to the genome data (Fig. 4), the most parsimonious explanation for the (Δ, a, α) genotype of Kaz E9 is the deletion of spacers 1028–1029 starting from the archetypal genotype (A, a, α). Analogous A-to-D transition events were confirmed in the Talas and Issyk-Kul regions in Kyrgyzstan and in the Samarkand region in Uzbekistan (Fig. 4). In all these cases, the previously proposed quick PCR approach around spacer 1029 (Kurz et al. 2021) failed to identify the proper ancestry of the strains with respect to their archetypal genotype.

Most Central Asian strains, except for KazE17, could be grouped in three discrete clusters that also displayed a certain geographical consistence (Fig. 3), whereas Georgian isolates formed two closely related clusters, i.e. genotypes (A, z, α) and (A, a, α)/(A, ä, α), within a clade comprising isolates from the Volga District and the Eastern Mediterranean area (Fig. 4).

## Discussion

Fire blight made its appearance in Kyrgyzstan and Kazakhstan in 2008, but the first reliable reports about its presence and distribution were published years later (Drenova et al. 2012; Djaimurzina et al. 2014). Even nowadays, due to the orographic fragmentation that characterizes the region and the remoteness of some areas, it is difficult to establish a comprehensive overview of the progression of the disease in the two countries. Likewise, little is known about the existing genetic diversity and where it originated from. The oldest *E. amylovora* isolates from the region examined so far dated back to 2012–2013 (Doolotkeldieva et al. 2021), roughly five years after the first detection of the disease, which provides only indirect indications about the original genotypes that were introduced into Central Asia.

Molecular characterization of most isolates was so far typically limited to PCR profiling of the CRRs (Doolotkeldieva et al. 2021; Gaganidze et al. 2021; Kurz et al. 2021; Sadunishvili et al. 2024), hence restricting the possibilities of recognizing the evolutionary relationships between different genotypes. Here, we filled this gap by increasing the number of genome-sequenced isolates and expanding the spatio-temporal frame of their origin. The genome data produced in this work with isolates from the seasons 2022–2023 are compatible with the characterization of earlier isolates from the same area obtained using a PCR approach (Doolotkeldieva et al. 2021).

Sequencing of the CRRs confirmed that genotype (Z, a, α) was the earliest and most widely distributed genotype in Kazakhstan, with the first case now documented in the region of Almaty in 2011. While this genotype was never detected in Kyrgyzstan, two variants based on the deletion of spacer 1029 were identified in the region of South Kazakhstan. It is worth remarking again that, in the PCR analysis, this specific deletion event in CRR1 results in the apparent shift of their archetypal genotype from A- to D-derived, even though the involved isolates clearly were originated from the A-lineage as it is obvious from the genome tree (Fig. 4).

The variability around spacer 1029 challenges the reliability of the previously proposed PCR approach targeting this spacer duplication for the attribution of isolates to one of the two ancestral populations that colonized Europe (Kurz et al. 2021) and can cause minor local genotype variations to appear like major population shifts. An example of this effect was also evident in a recent study on the distribution of *E. amylovora* lineages in northern Italy (Albanese et al. 2022), in which the loss of one of the two copies of spacer 1029 caused the reversion to genotype (D, a, α) in a confined group of isolates that were embedded within a population carrying the geographically more widespread genotype (A, a, α), according to the related core genome tree (Albanese et al. 2022). Nonetheless, considering the position in the genome tree of European strains belonging to the archetypal genotype (D, a, α) isolated previously (Fig. 4), the conclusions of the study by Kurz et al. (Kurz et al. 2021) regarding the early phases of the colonization of Europe by two distinct populations of *E. amylovora* still seem to hold general validity.

Widely distributed isolates in Central Asia belong to the genotype (A, t, α) and its derivative (A, u, α), which were detected in contiguous regions of Kazakhstan (Zhambyl and Almaty regions) and Kyrgyzstan (Chui Valley and the surroundings of lake Issyk-Kul) as of 2012, suggesting that they belonged to a separate early introduction event, which then spread transnationally. Both genotypes were still detected by the most recent surveys in the affected areas. Genotype (A, t, α) and its derivative (Ã, t, α) were detected as well in the genomes of two isolates that were recently documented from the neighboring Xinjiang Uygur Autonomous Region (Fei et al. 2023a, b). Here, fire blight was first reported in 2016, thus possibly indicating an outflow of the disease from Central Asia toward China.

Surveys in other regions of Kyrgyzstan revealed the presence of local genotypes that were not found elsewhere to date. In the Jalal-Abad region, two different genotypes were retrieved, i.e., (A, å, α) and (A, ë, α), both are the independent derivatives of archetypal genotype (A, a, α). Another modification of the latter is the A-derived genotype (Л, a, α), which was detected along to (D, a, α)-derived genotype (D, б, α) in the Talas region. The presence of isolates apparently belonging or derived from the D-genotype in Central Asia was not reported in previous studies (Doolotkeldieva et al. 2021; Kurz et al. 2021), but could be repeatedly detected in this study across several geographically separated sampling sites. Genotype (D, ʎ, α) was identified in an isolate retrieved from the surroundings of lake Issyk-Kul, while genotype (D, a, α) was retrieved both in surroundings of Almaty (Kazakhstan), which is also the area where the highest number of different genotypes was detected, as well as in the region of Samarkand in Uzbekistan, where the archetypal genotype (A, a, α) was also detected. Comparative genomic analysis (Fig. 4) suggested that the apparent presence of D-derived genotypes in Central Asia is attributable to local genotype variation (independent loss of spacer 1029) rather than to separate introduction events in the country. Similarly, genotype (B, t, α), characterizing strain TaE1 from the Shahrinav district in Tajikistan, is compatible with the deletion of spacer 1022 from genotype (A, t, α) (Fig. 2), despite the geographical distance to the genomically related Kazakh and Kyrgyz isolates carrying the latter genotype.

As no official record for both countries is present in the Global Database of the European and Mediterranean Plant Protection Organization (EPPO) (https://gd.eppo.int/taxon/ERWIAM/distribution), this is, to our knowledge, the first report emphasizing the presence and the genetic characteristics of fire blight isolates from Uzbekistan and Tajikistan.

In comparison to the genetic diversity encountered in Europe, an accelerated diversification of the CRRs is apparent in Central Asia, a trend that is also confirmed by the increasing number of amino acid substitutions in genome analysis (Fig. 4). Within the first three decades after its introduction in Europe, practically no variation in the CRRs was observed in *E. amylovora*, and identical genotypes could still be found all over the continent (Kurz et al. 2021). On the contrary, the number of genotypes escalated considerably in the limited time after the disease arrived in Central Asia. The reason for this trend is unclear, but it could be revealing to an adaptation process to new environmental conditions and the larger genetic diversity of the host in its center of origin.

## Conclusion

To summarize, we were able to confirm the prevalence of three main population groups of *E. amylovora* in Central Asia using the complete sequences of the CRRs and genome sequence analysis, with the presence of cluster Za in all three investigated Kazakh regions, and cluster (A, t, α) not only extending between southeast Kazakhstan and northeast Kyrgyzstan but leaking into China. All other genotypes displayed a more discrete distribution that was limited only to certain areas. Genotypes (A, a, α) and (D, a, α) were the only ones retrieved in Central Asia for which a match was found elsewhere, but genomic analysis revealed that only genotype (A, a, α) was directly derived from the identical ancestral genotype that was introduced into Europe, while the presence of genotype (D, a, α) is due to a *de novo* deletion of one of the two copies of spacer 1029. *E. amylovora* isolates from Central Asia were genetically most closely related to isolates from Iran and the Middle East, while Georgian isolates clustered within a group containing strains from the Volga District (Russia) and the East Mediterranean area, indicating the possible introduction patterns (Fig. 4).

We have further shown here that spacer 1029 is a hotspot of variability and that the transversion from A- to D-derived genotypes has occurred on few occasions in the modern evolutionary history of *E. amylovora*, possibly blurring the corresponding phylogenetic signal and thus making the proposed PCR approach (Kurz et al. 2021) less reliable, especially when analyzing more recent isolates. However, within restricted geographical areas and especially during the initial phases of an epidemic, the implementation of PCR assays targeting locally-relevant CRISPR genotypes remains a useful tool for preliminary diversity analysis and the selection of the isolates to be sequenced through NGS technologies for in-depth phylogenetic investigation (Doolotkeldieva et al. 2021; Sadunishvili et al. 2024).

## Methods

### Selected 2020–2022 sampling sites

Field inspections for fire blight were conducted in Kyrgyzstan and Georgia during the 2021–2022 and 2020–2022 seasons, respectively. In Kyrgyzstan, three regions had already been scrutinized during the 2018–2019 campaigns, i.e., Issyk-Kul, Chui Valley, and Jalal-Abad (Doolotkeldieva et al. 2021), while a fourth, the Talas region, which is located in north-west of Kyrgyzstan on the slopes of the Ala-Too mountain range, was included for the first time in this survey. The area has a mid-temperate continental climate, with warm summers and cold winters. The average temperature is 6–8°C, and the yearly precipitation is about 320 mm, mainly concentrated during the crop-growing season (late spring). In Georgia, surveys were conducted during the 2020–2022 seasons in orchards of the four pome-fruit growing regions in the East of the country, i.e., Mtskheta-Mtianeti, Shida Kartli, Kvemo Kartli, and Kakheti (Sadunishvili et al. 2024). Isolates from the neighboring countries covering the period 2013–2017 were provided by the All-Russian Plant Quarantine Center (VNIIKR) and originated from all fruit-growing regions of Kazakhstan (Zhambyl, South Kazakhstan, and Almaty region), from the region of Samarkand in Uzbekistan, and from the Shahrinav District in western Tajikistan. All isolates were recovered from aerial parts of the plant showing clear fire blight symptoms and were identified as *E. amylovora* following the standard EPPO protocols (Anonymous 2022). Considering the strains already included in previous publications and characterized by different approaches (Djaimurzina et al. 2014; Drenova et al. 2014; Bühlmann 2015; Doolotkeldieva et al. 2021; Fei et al. 2023a, b), we analyzed herein the genetic diversity of a total of 86 *E. amylovora* strains isolated from diseased fruit trees from the *Amygdaloideae* family across the Eurasian continent, plus a reference strain from the United States (Table 1).

### Genome sequencing and analysis of the CRISPR repeat regions (CRRs)

Whole-genome sequencing (WGS) of 27 new isolates from Central Asia and Georgia was performed using the Illumina approach following the standard manufacturer’s protocol as described before (Pothier et al. 2022). The isolates were selected based on the genetic polymorphism determined through PCR assays targeting specific spacer regions within the CRISPR arrays (Doolotkeldieva et al. 2021; Kurz et al. 2021; Sadunishvili et al. 2024) and/or on criteria of geographic and temporal diversity. Genomes were assembled using unicycler (v0.4.8) (Wick et al. 2017) and annotated using Prokka (v1.4.2) (Seemann 2014). Fifty previously sequenced genomes from the literature were also included in the analysis (Table 1).

The core genome tree was generated from a private EDGAR3.0 project (Dieckmann et al. 2021) containing both published and unpublished genomes of *E. amylovora* (Bühlmann 2015; Smits et al. 2017), together with the genomes sequenced in this study. Briefly, the core genome was computed for the selected isolates, after which alignments of each core gene set were generated using MUSCLE (Edgar 2004), and concatenated to one alignment. This was subsequently the input for tree construction using the FastTree software (Price et al. 2010) to generate approximately-maximum-likelihood phylogenetic trees. The local support values were determined using the Shimodaira-Hasegawa test. Values below 50 were removed.

The CRISPR regions of ten additional isolates not included in the WGS set were characterized by implementing the previously described primer crawling strategy based on the traditional Sanger sequencing approach (Rezzonico et al. 2011) (Table 1). CRISPR spacers and repeats were identified by analyzing the assembled sequences in CRISPRCasFinder (Couvin et al. 2018) and spacers were manually aligned in MS Excel to those of known genotypes in accordance with the nomenclature proposed earlier (Rezzonico et al. 2011).

## Data Availability

The datasets used and/or analyzed during the current study are available from the corresponding author on reasonable request.

## Abbreviations

CRISPR: Clustered regularly interspaced short palindromic repeats
CRR: CRISPR repeat region
EPPO: European and Mediterranean plant protection organization
IUCN: International union for conservation of nature
NGS: Next generation sequencing
PCR: Polymerase chain reaction
WGS: Whole-genome sequencing
WP: Widely-prevalent
